# A Comparison of Statistical Methods for Identifying Out-of-Date Systematic Reviews

**DOI:** 10.1371/journal.pone.0048894

**Published:** 2012-11-20

**Authors:** Porjai Pattanittum, Malinee Laopaiboon, David Moher, Pisake Lumbiganon, Chetta Ngamjarus

**Affiliations:** 1 Department of Biostatistics and Demography, Faculty of Public Health, Khon Kaen University, Khon Kaen, Thailand; 2 Clinical Epidemiology Program, Ottawa Hospital Research Institute, Ottawa, Ontario, Canada; 3 Department of Epidemiology and Community Medicine, Faculty of Medicine, University of Ottawa, Ottawa, Ontario, Canada; 4 Department of Obstetrics and Gynaecology, Faculty of Medicine, Khon Kaen University, Khon Kaen, Thailand; University of Michigan, United States of America

## Abstract

**Background:**

Systematic reviews (SRs) can provide accurate and reliable evidence, typically about the effectiveness of health interventions. Evidence is dynamic, and if SRs are out-of-date this information may not be useful; it may even be harmful. This study aimed to compare five statistical methods to identify out-of-date SRs.

**Methods:**

A retrospective cohort of SRs registered in the Cochrane Pregnancy and Childbirth Group (CPCG), published between 2008 and 2010, were considered for inclusion. For each eligible CPCG review, data were extracted and “3-years previous” meta-analyses were assessed for the need to update, given the data from the most recent 3 years. Each of the five statistical methods was used, with random effects analyses throughout the study.

**Results:**

Eighty reviews were included in this study; most were in the area of induction of labour. The numbers of reviews identified as being out-of-date using the Ottawa, recursive cumulative meta-analysis (CMA), and Barrowman methods were 34, 7, and 7 respectively. No reviews were identified as being out-of-date using the simulation-based power method, or the CMA for sufficiency and stability method. The overall agreement among the three discriminating statistical methods was slight (Kappa = 0.14; 95% CI 0.05 to 0.23). The recursive cumulative meta-analysis, Ottawa, and Barrowman methods were practical according to the study criteria.

**Conclusion:**

Our study shows that three practical statistical methods could be applied to examine the need to update SRs.

## Introduction

Systematic reviews (SRs) are an important scientific tool that can provide accurate and reliable evidence, typically about the effectiveness of health interventions [1]. SRs are a useful starting point for practice guideline developers, health policy analysts, and health care providers [1], [2]. A few granting agencies are starting to require SR evidence when making decisions about funding new research, particularly randomized control trials [1], [3].

Recent data suggest that 11 new SRs are published daily, while annually 5,000 SRs are indexed in Medline [4]. SRs are most useful when they are up-to-date. Evidence is dynamic, and if SRs are out-of-date this information may not only be unhelpful, it may be harmful. In 1995 Jadad et al [5] reported that only 3% of the 39 non-Cochrane SRs and 50% of 36 Cochrane SRs had been updated 2 years after publication. In 2002, 70% of 362 Cochrane SRs published in 1998 had been updated [6]. Only 2% (2/88) of non-Cochrane SRs and one-third (47/125) of the Cochrane SRs (focusing on therapeutic effectiveness) were updated in 2004 [7]. Garrity et al. [8] conducted an internet-based international survey of healthcare organizations involved in SRs, and found that only 33% (35/105) of respondents updated their SRs regularly, although most of them agreed with the importance of updating SRs.

We built on a recent systematic review of strategies, techniques, and statistical approaches on when and how to update SRs [9], [10], searching for further information about statistical methods to update SRs. At present there are five proposed statistical methods to determine whether a given SR is out-of-date (see Table 1 for more details):

1. recursive cumulative meta-analysis (CMA); [11]
2. CMA for sufficiency and stability; [12]
3. a test for identifying null meta-analyses that are ripe for updating (Barrowman method); [13]
4. quantitative signal of changes in evidence (Ottawa method) [14]; and
5. the power of an updated meta-analysis using simulation (simulation-based power method) [15].

**Table 1:** Five statistical methods for identifying out-of-date reviews.

Method	Details	Indicator(s) of an out-of-date review	Strengths	Limitations
Recursive CMA [11]	A relative change in treatment effect of at least 50%. The relative change = pooled treatment effect from the updated meta-analysis/pooled treatment effect from the current meta-analysis.	Relative change ≤0.5 or ≥1.5	Simple calculation relative change in treatment effectsThe relative change may signal biases or heterogeneity among included studies, if the ratio is substantially different from 1	The cut-off criteria of 0.5 and 1.5 are subjective and arbitrary.The relative change will tend to be unstable for small treatment effects.
CMA for sufficiency and stability [12]	Two indicators, sufficiency and stability, are used to consider whether the SR is out-of-date. Sufficiency is measured as the failsafe ratio, calculated as N_fs_/(5k+10), where N_fs_ = 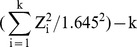 Z_i_ is the pooled standardized treatment effect of the previous meta-analysis, while k is number of included studies in the previous meta-analysis. Stability is measured as the slope of the linear regression fitted across the cumulative treatment effects calculated from included studies of the updated meta-analysis, versus information increment (number of included studies).	failsafe ratio >1 and absolute slope of the linear regression >0	Robustness from consideration of two indicators: the potential number of unretrieved studies (sufficiency); and the slope of cumulative treatment effects (stability)	Potential autocorrelation arises because the errors associated with the data points for the linear regression may not be independent.
Barrowman method [13]	The participant ratio (q) was calculated from q = m/n where m is the observed number of participants in the study(ies) published within the most recent 3 years, and n is the expected number of participants in the study(ies) published within the most recent 3 years, 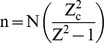 Z_c_ is the critical value of the Z-statistic for the desired probability value (e.g., 1.96 for a two-sided type I error rate of 0.05)Z (denominator) is substituted with the standardized treatment effect of the previous meta-analysis, and N is the total number of participants.	Participant ratio (q)>1	q is a straightforward calculation.All elements for calculation are generally provided in meta-analysis software packages	Limited to the original meta-analysis with statistically non-significant results
Ottawa method [14]	The two quantitative signals were considered;(i) Change in statistical significance (note: 0.04<p<0.06 range not considered sufficient signal).(ii) Change in effect size of at least 50% (relative risk reduction (RRR) of updated meta-analysis, to RRR of previous meta-analysis, were calculated for treatment effects measured as a relative ratio (RR, OR). For mean difference (MD), the relative change was calculated as the recursive CMA.	Significant updated meta-analysis; and/or ratio of RRR≤0.5 or RRR≥1.5. or ratio of MD≤0.5 or ratio of MD≥1.5	Robust, because indicator (i) ignores trivial changes by restricting the p-value of updated meta-analysis <0.04 instead of <0.05	Change in effect size of at least 50% is arbitrary
Simulation-based power method [15]	The simulation technique was used to generate a new study data based on the estimated parameters yielded from the 3-year previous data, and the included study(ies) published within 3 years of the most recent study. The new study data was added to the previous meta-analysis and then re-meta-analyzed. The hypothesis testing for the pooled treatment effect at 5% significant level was conducted. The new study data was simulated repeatedly for 10,000 times, and then calculated the power – proportion of significant result from those 10,000 re-meta-analyses. The power>80% indicated that the given SR was out-of-date. (*Details are presented in Appendix S1*).	Power≥80%	Tends to produce more accurate results, using a simulation technique with many iterations	Requires skill in statistical programming

*CMA = cumulative meta-analysis; MD = mean difference; n = number in a subgroup; N = number in a cohort, or total number in a study; OR = odds ratio; RR = relative risk; RRR = relative risk reduction; Z = Z statistic.*

With no standard approach, it is unclear when and how to update SRs. The Cochrane Collaboration advocates periodic updating every 2 years [16], [17]. This updating strategy may not be appropriate for all SRs; in one study examining 100 SRs, 23% of 100 SRs became out-of-date within 2 years after publication, 15% within one year, and 7% were already out of date at the time of publication [14]. One contributory reason is possibly different publication trajectories for different treatments, and/or various conditions. Also of concern is that simulation studies suggest that frequent updating of SRs can result in an inflated type I error rate [18], and might lead to publication bias [10] because the studies with significant results are likely to be published faster than those with non-significant results.

Of the available statistical methods for updating, among 99 respondents to the aforementioned Internet-based survey, 11% used a CMA method (not specified which of the two), while 4% favored the Barrowman method [8]. One recent study [19] found that the Barrowman and simulation-based power methods produced results that agreed closely, when the study sample was homogeneous. There is no further information regarding other statistical methods.

Since there are five statistical methods available to identify whether a SR is out of date, and there is no evidence comparing these methods in terms of their agreement with one another, consistency nor practicality, we aimed to compare these methods for identifying out-of-date SRs.

## Materials and Methods

A sample of SRs registered in the Cochrane Pregnancy and Childbirth Group (CPCG) published between 2008 and 2010 in the Cochrane Database of Systematic Reviews (CDSR) was examined. We selected the CPCG because it was the first Cochrane Review Group to be established, and it has published the largest number of Cochrane SRs.

### Primary outcome identification

For the purposes of this study, a single primary outcome was identified for each study as the outcome of interest.

- When the CPCG review authors defined a single primary outcome, that one was used.
- If the review authors pre-defined more than one, the outcome with the largest number of included studies and/or participants was chosen as the primary outcome of interest. If more than one primary outcome satisfied these criteria, an obstetrician (Pisake Lumbiganon; PL) selected the single most clinically important one.
- If no primary outcome had been identified by the review authors, the reported outcomes were ranked by PL, based on clinical importance and the CPCG review's objective.

### Inclusion criteria

The CPCG review was included if it met the following inclusion criteria:

- it reported a meta-analysis of at least 3 included studies for the primary outcome;
- the analysis did not include results of cluster randomized trials;
- the primary outcome measure was either dichotomous or continuous, and
  - For a dichotomous outcome, numbers of events and sample sizes in treatment and control groups were reported
  - For continuous outcomes, the means, standard deviations and sample sizes in treatment and control groups were reported
- the publication date of the most recent included study was at least 3 years later than dates of the first two included studies
- the included studies that were published at least 3 years before the most recent included study, yielded a non-significant meta-analysis at the 5% significance level.

### Searching and selection

A list of all active CPCG review titles was identified through Archie – the Cochrane Collaboration's central server (accessed April, 2011). The reviews were retrieved for full text from the Cochrane Library and screened according to defined inclusion criteria.

### Data extraction

A data collection form was used to extract data from the eligible CPCG reviews (e.g., topics in obstetrics, study objective, primary outcome, and comparisons). For individual studies included in each CPCG review, the year of publication and summary statistics were also extracted, as indicated in inclusion criteria above.

One author (Porjai Pattanittum; PP) extracted the data from all eligible CPCG reviews. To check data extraction accuracy, data from a random sample of 10% of the reviews was extracted independently by a second reviewer (Chetta Ngamjarus; CN). The rate of discrepancies was 0.125 (1/8 reviews); this rate was very small (1/80 items) if considered by item. Discrepancies on data extraction and errors were resolved by rechecking with the full text of PCG reviews.

### Statistical methods for identifying an out-of-date SR

To examine the detection of out-of-date SRs using each method, we compared the results of a previous meta-analysis and an updated meta-analysis. The previous meta-analysis was defined as a meta-analysis of the studies published more than 3 years before the most recent study (this was a hypothetical review), while the updated meta-analysis including all studies, and was the actual CPCG review.

We selected a 3 year period between the previous meta-analysis and updated meta-analysis in part because this period is one of the criteria for one statistical method (the simulation-based power method [15]) examined in this present work. As well, Jaidee et al [20] reported that a median time before the first update of CPCG reviews was 3.3 years (95% CI 2.7 to 3.8 years).

The cohort of eligible CPCG reviews was assessed for the need to update using the five statistical methods [11], [12], [13], [14], [15]. Methods, and their strengths and limitations are briefly summarized in Table 1, while more details are available elsewhere [9], [10].

A random effects model was used, as a conservative method for meta-analysis of results [21].

### Outcome measures and data analysis

The main outcomes of this study were agreements between methods as to the need for updating, as well as assessment of the ease of calculations and practicality of each method.

We calculated the agreement in identifying out-of-date SRs among all possible study methods by a pooled Kappa statistic and its corresponding 95% confidence interval (CI). Frequencies and survival time to update with 95% CI were used to describe characteristics of the study cohort. Data analyses were conducted using R software [22] and STATA [23].

## Results

**Figure 1:**
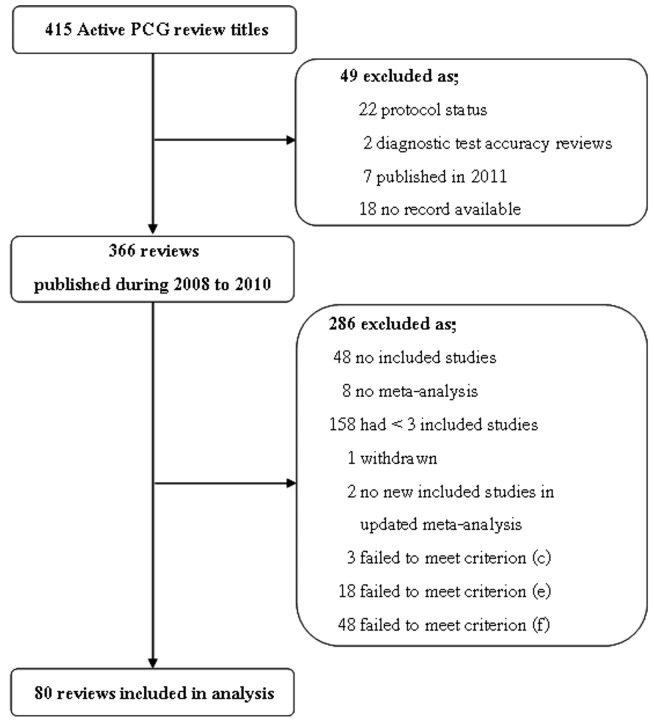
Flow diagram indicating results of Cochrane PCG reviews with inclusion and exclusions.

Our study assessed out-of-date in the null meta-analyses of CPCG reviews. The search identified 415 active review titles, which were screened as depicted in Figure 1 Ultimately, 80 reviews were included.

### Characteristics of included CPCG reviews

Among the 80 reviews, 55% were published in 2010, and 95% reported the primary outcome as dichotomous data, of which 89% presented treatment effects as risk ratios. The median numbers of included studies before and after updating were 4 and 5.5 studies, respectively. The median numbers of participants before and after updating were 1,346, and 2,274 persons, respectively (Table 2). The most common review topic was induction of labour (14/80). Sixty percent (48/80) of the reviews had been updated previously; up to three times. Of the 48 Cochrane updated reviews, 8 reported that the conclusion had changed after the update activities.

**Table 2:** Characteristics of CPCG reviews (N = 80).

Characteristic	Statistics
**Year of publication of most recent review - Number (%)**	
2008	7 (8.8)
2009	29 (36.2)
2010	44 (55)
**Primary outcome data - Number (%)**	
Dichotomous	76 (95)
Continuous	4 (5)
**Estimate of treatment effect - Number (%)**	
Risk ratio (RR)	71 (88.8)
Odds ratio (OR)	5 (6.2)
Mean difference (MD)	4 (5)
**Median of included studies before updating (q1 ; q3)**	4 (2 ; 8.5)
**Median of included studies after updating (q1 ; q3)**	5.5 (4 ; 10)
**Median of participants before updating (q1 ; q3)**	1,346 (429 ; 3,116)
**Median of participants after updating (q1 ; q3)**	2,274 (797 ; 5,723)
**Updating – Median time to most recent update (95% CI)**	
First report (32 SRs)	NA
First update (27 SRs)	6.9 (4.3 to 8.3)
Second update (17 SRs)	4.7 (3.4 to 7.7)
Third update (4 SRs)	0.87*

* *due to the small number of reviews, the 95% CI cannot be estimated.*

*q1 ; q3 = interquartile range; CI = confidence interval; NA = not applicable.*

### Comparing out-of-date detection using the five methods

Applying the five statistical methods to the 80 reviews, the Ottawa method identified 34 reviews (Appendix S4; 5, 8, 10, 12,14,16, 18, 20, 23, 25–30, 32–34, 39–40, 44, 46–47, 49, 52–53, 58–59, 61, 67, 72, 74, 78–79) as being out-of-date, while the recursive CMA and Barrowman methods each identified 7 reviews as being out-of-date. The CMA for sufficiency and stability, and the simulation-based power method did not identify any review as being out-of-date. Brief results of each method are presented for the 10 reviews with the highest magnitude of the indicators in Appendix S3, Table S3 to S7.

### Recursive CMA method

Seven of the 80 reviews yielded a signal indicating a need for updating: 4 reviews had an out-of date ratio greater than 1.5; the remaining 3 reviews produced a ratio less than 0.5 (Appendix S3, Table S3). Of these, 3 reviews presented changes in directions of treatment effects in updated meta-analyses.

### CMA for sufficiency and stability method

All of 80 reviews yielded a failsafe ratio less than 1, which could imply that too few additional studies were available to update the previous meta-analysis. The stability of effect size could not be explored because too few studies were identified in the three year interim period.

In Appendix S3, Table S4 it can be seen that the number of ‘hidden’ study(ies) (N_fs_) was smaller than the benchmark (e.g., Abalos E, 2007 (Appendix S4; 1) revealed N_fs_ = 20 studies but the benchmark was 110 studies, with a failsafe ratio = 0.18). As a result of lack of sufficiency to determine the stability of effect size, the out-of-date status of none of the 80 reviews could be predicted using this method.

### Barrowman method

Seven of 80 reviews were deemed to be out-of-date using this method. The highest participant ratio was 34.9, with the treatment effect measured as mean difference (MD). The results of this method were shown in Appendix S3, Table S5. Although the participant ratios of those 7 reviews identified as being out-of-date exceeded unity, only 2 reviews (Appendix S4; 20, 32) provided significant results for the updated meta-analyses (not shown in Table S5).

### Ottawa method

This method indicated 34 (43%) reviews as being out-of-date. Three reviews were detected by the first quantitative signal (change in statistical significance), and 31 reviews were found by the second quantitative signal (change in effect size of at least 50%). The maximum and minimum RRR or MD ratios were 33.1 and −15.5. Thirty-one reviews reported relative risk, while 3 reported mean difference. Ten reviews (Appendix S4; 18, 29–30, 34, 39, 44, 53, 58, 59, 78) presented changes in the direction of the treatment effect compared with the results of previous meta-analyses (not shown in Appendix S3, Table S6).

### Simulation-based power method

No review was identified as being out-of-date using this method. The maximum power of update meta-analysis was only 63% (Appendix S3, Table S7).

### Agreement between methods

Thirty-seven reviews were identified to be out-of-date by one or more statistical methods; recursive CMA, Barrowman, and Ottawa methods with slight agreement between them (Kappa = 0.14; 95% CI 0.05 to 0.23). Only one review (Appendix S4; 25) was identified as out-of-date by all three methods.

Among the three pairs of methods that discriminated between reviews potentially needing updating in this work, the observed agreement ranged from 43 to 69 reviews not needing updating, while the positive results ranged from 3 to 5 reviews. Fair agreement was observed between the recursive CMA and Barrowman methods (Kappa = 0.37; 95% CI 0.03 to 0.72; see Appendix S3, Table S8).

### The practicalities of methods

Practical methods were considered to be those requiring less intensive analysis, and the straightforward data. The recursive CMA and the Ottawa methods were the most practical methods because they require only two parameters to calculate the indicator (pooled treatment effects from current and updated meta-analyses, or p-value from current and updated meta-analysis). These parameters are automatically calculated by any meta-analysis software. The Barrowman method also does not require the updated meta-analysis to be performed; only sample sizes and Z-statistics from additional studies are required.

## Discussion

There is an increasing number of statistical methods aimed at detecting signals of the need to update systematic reviews. Comparing five of these approaches, three methods could detect potentially out-of-date SRs in our sample of 80 CPCG reviews.

A cut-off of relative change in treatment effect of either <0.5 or >1.5 for the recursive CMA is arbitrary, and seems quite large. This method detected five more out-of-date reviews (Appendix S4; 32, 45–46, 49, 73). With a narrower up-to-date range of less than 25% change; the arbitrary cut-off point, a total of 12 out-of-date reviews were identified.

The CMA sufficiency and stability method represents the most stringent test to detect an out-of-date review; none of 80 reviews had a sufficient number of new studies. The average additional study(ies) in the updated SRs in our sample was two studies, whereas the CMA sufficiency and stability method requires six studies to overturn the significance in meta-analysis (N_fs_). The six hidden studies are, however, a much smaller number than the average benchmark (41 studies), which is why all 80 reviews had a sufficiency below unity. Conversely, the Ottawa method is a sensitive method to detect a potentially out-of-date review. Thirty-four reviews were predicted to be out-of-date according to this method. Although the simulation-based power method detected no out-of-date reviews with a power of at least 80%, sensitivity, is increased at lower powers, and this method would identify 4 reviews as being out-of-date when the power was at least 60%.

Our findings show fair agreement between the recursive CMA and Barrowman method in identifying out-of-date systematic reviews. Sutton et al [19] compared two statistical methods, the Barrowman method and simulation-based power method, across 12 reviews and found the Barrowman method identified 5 reviews as being out-of-date, while only one review was detected by the simulation-based power method. With our sample of reviews the Barrowman method identified seven (of 80) reviews as out-of-date, while the simulation-based power method did not identify any out-of-date review. The review identified by the simulation based power method in Sutton et al [19] presented the highest power at 89%, p-value after updating was 0.01. This case added 7,397 participants in 5 studies, in addition to the original 7 studies. In our study, the highest power was 63.4%, with a p-value after updating of 0.21. Only a single additional study was added to the previous two studies. The 377 additional participants were observed and this was only 5% (377/7,397) compared to the Sutton study.

A recent study [24] compared the Ottawa method (using modified qualitative signal, and quantitative signal) to the RAND method (a combination of literature search and the assessment of content experts) across four systematic reviews, 77 outcomes. The paper reported substantial agreement between the methods (Kappa = 0.64, 95% CI 0.45 to 0.83). Our study found less agreement between methods. A possible reason is that the study of Shekelle, et al [24] applied both quantitative and qualitative approaches, whereas our study only used quantitative signal for the need to update SRs.

In further exploration of the 37 reviews that had been identified for the need to update by one or more statistical methods, we compared the features of the results from our analyses with the eight updated reviews in which the conclusions changed in The Cochrane Library. Of the 34 out-of-date reviews detected by the Ottawa method, 3 had changes in their conclusions in the updated Cochrane report. The Barrowman method indicated 7 outdated reviews, 3 of which had changed conclusions in the updated Cochrane report. None of seven out-of-date reviews identified by the recursive CMA method had changed their conclusions. Only 2 reviews with changed conclusions (Appendix S4; 30, 52) included the same study(ies), comparison and outcome as in the present work. Upon closer examination of the 8 updated Cochrane reviews with changed conclusions it was apparent that discrepancies arose due to differences in the updating time periods, resulting in mismatches between studies included in our and Cochrane “previous” meta-analyses.

With a low power to detect out-of-date reviews, and due to the study design not matching updating periods in The Cochrane Library, there were few out-of-date reviews identified by the Ottawa and Barrowman methods, and none by the recursive CMA method that corresponded with changed conclusions after updating in The Cochrane Library. Further research would ideally use a prospective data collection and analyses to flag reviews that are potentially out of date using a range of statistical methods, and correlate the predictions with subsequent changes in conclusions following updating.

Limitations of this work include the aforementioned study design. As well, we applied the five statistical methods in a retrospective 80 reviews with non-significant meta-analysis at 5% significant level by using a made up updating time – removing the most recent 3 years of included study (ies). The number and types of reviews meant that we could not explore subgroups such as agreement between methods according to the type of effect measure (none of the reviews reported treatment effects as relative difference, or standardized mean difference), and the study cohort was restricted to CPCG reviews.

The practical methods (recursive CMA, Ottawa, and Barrowman methods) suggested by this study can be used for surveillance of the need to update systematic review. However, there is currently no standard approach to determining if a SR is in need of updating. The statistical methods examined in this study were not consistent with one another, in some cases at most agreeing slightly. These methods are all based on changes in statistical significance and precision, which do not take into account other important factors such as an emergence of a superior alternative treatment, or new information on benefit or harm of treatment that contribute to a decision to update, as well as the potential risk of bias(es) of the new evidence from trial(s). Thus our findings represent additional information, rather than a solid basis for the decision.

## Supporting Information

Appendix S1 **An approach to calculate the power of updated meta-analysis.**
(DOC)

Appendix S2 **Formula for estimating probability of an event, and mean in the treatment arm.**
(DOC)

Appendix S3 **Table S3 to S8.**
(DOC)

Appendix S4 **References of the cohort of CPCG reviews.**
(DOC)
