# Supplementary material for: A Comparison of Statistical Methods for Identifying Out-of-Date Systematic Reviews
Source: PLoS One. 2012 Nov 20;7(11):e48894. doi: 10.1371/journal.pone.0048894 (PMC3502410; doi:10.1371/journal.pone.0048894)
Supplement: Appendix S1 — An approach to calculate the power of updated meta-analysis. (DOC) [file pone.0048894.s001.doc]

**Appendix S1:**

**An approach to calculate the power of updated meta-analysis**

The simulation-based power method [15] estimates the “power probability,” for deciding whether the review is out of date. The procedure was as follows:

(a) Estimate parameters for a new study data

The treatment effect of a new study was sampled from the t-distribution with the parameters yielded from the 3-year previous data; the pooled treatment effect and its variance.

For dichotomous outcome, an average probability of an event across the studies in the control group from the 3-year previous data was used as the probability of an event in the control group of the new study. An estimate of the event rate in the treatment group can be derived (see Appendix S2).

For continuous outcomes, the mean and standard deviation of the new study in the control group was estimated by using an unweighted average mean and unweighted standard deviation across the studies in the control group from the 3-year previous data. The mean and standard deviation of the new study in the treatment group can be derived (see Appendix S2).

The number of participants of each arm the new study was equal to the total number of participants of each arm in the included study(ies) published within 3 years of the most recent study.

(b) Simulate the new study data

The estimated parameters in (a) were used to simulate the new study data using a binomial distribution for the dichotomous data, and using a normal distribution for the continuous data.

(c) Re-meta-analyze including new study

Added the new study data to the previous meta-analysis and re-analysis of the pooled treatment effect; called “a simulated update meta-analysis”.

(d) Hypothesis test

The pooled treatment effect from the simulated update meta-analysis was tested as to whether it was significantly different from null at 5% significant level.

(e) Calculate the power

Steps (b) to (d) were repeated 10,000 times, the proportion of significant result from (d) was calculated, and called “power”. Power >80% indicated that the given SR was out-of-date.
