# Supplementary material for: A Comparison of Statistical Methods for Identifying Out-of-Date Systematic Reviews
Source: PLoS One. 2012 Nov 20;7(11):e48894. doi: 10.1371/journal.pone.0048894 (PMC3502410; doi:10.1371/journal.pone.0048894)
Supplement: Appendix S2 — Formula for estimating probability of an event, and mean in the treatment arm. (DOC) [file pone.0048894.s002.doc]

**Appendix S2**

**Formula for estimating probability of an event, and mean in the treatment arm**

i) the probability of an event in the treatment arm for RR

$P_{trt}$ = . $P_{ctrl}$

ii) the probability of an event in the treatment arm for OR

$P_{trt}$ =

iii) the mean in the treatment arm for MD

$\overline{X}_{trt}$ = MD$+\overline{X}_{ctrl}$

*Note*: RR = Risk ratio; OR = Odds ratio; MD=Mean difference;

P_trt_ , P_ctrl_ = the probability of an event in the treatment , and control groups;

$\overline{X}_{trt}$, $\overline{X}_{ctrl}$= sample mean in the treatment, and control groups
