# Supplementary material for: A Comparison of Statistical Methods for Identifying Out-of-Date Systematic Reviews
Source: PLoS One. 2012 Nov 20;7(11):e48894. doi: 10.1371/journal.pone.0048894 (PMC3502410; doi:10.1371/journal.pone.0048894)
Supplement: Appendix S3 — Table S3 to S8. (DOC) [file pone.0048894.s003.doc]

**Appendix S3**

**Table S3.** Results of applying the Recursive CMA method

| **Article identifier*** | **Measure of TE** | **Number of included studies_previous_** | **TE_previous_** | **Number of included study_updated_** | **TE_updated_** | **Ratio** | **Predicted Status** |
| --- | --- | --- | --- | --- | --- | --- | --- |
| Young, 2002 | OR | 2 | 0.12 | 3 | 0.31 | 2.62 | out-of-date |
| Kramer, 2002 | MD | 3 | 3.19 | 4 | -7.95 | -2.49 | out-of-date |
| Rumbold, 2005 | RR | 2 | 0.23 | 4 | 0.53 | 2.25 | out-of-date |
| Dodd, 2008 | RR | 2 | 0.64 | 5 | 1.06 | 1.67 | out-of-date |
| Crepinsek, 2010 | RR | 2 | 0.27 | 3 | 0.45 | 1.66 | out-of-date |
| Crowther, 2007 | MD | 2 | -137.67 | 4 | -62.07 | 0.45 | out-of-date |
| Mahomed, 2007 | MD | 11 | 11.41 | 14 | -2.61 | -0.23 | out-of-date |
| *Hofmeyr, 1998* | *RR* | *2* | *0.37* | *3* | *0.54* | *1.45* | *not out-of-date* |
| *Thomas, 2001* | *RR* | *4* | *0.79* | *5* | *1.11* | *1.41* | *not out-of-date* |
| *Duley, 2006* | *RR* | *2* | *0.24* | *3* | *0.33* | *1.38* | *not out-of-date* |

***** *Appendix S4; 80, 53, 68, 29, 21, 25, 59, 45, 73, 32*

*TE = treatment effect*

**Table S4.** Results of applying the CMA for Sufficiency and stability method

| **Article identifier*** | **Measure of TE** | **Number of included studies_previous_** | **Number of included studies_updated_** | **N_fs_** | **Benchmark** | **failsafe ratios** | **Predicted Status** |
| --- | --- | --- | --- | --- | --- | --- | --- |
| Boulvain, 2008 | RR | 24 | 26 | 23.7 | 130 | 0.18 | NA |
| Kelly, 2009 | RR | 22 | 33 | 21.7 | 120 | 0.18 | NA |
| Alfirevic, 2006 | RR | 22 | 25 | 21.3 | 120 | 0.18 | NA |
| Abalos E, 2007 | RR | 20 | 22 | 20.0 | 110 | 0.18 | NA |
| Alfirevic, 2009 | RR | 21 | 24 | 19.7 | 115 | 0.17 | NA |
| Kenyon, 2010 | RR | 16 | 18 | 15.8 | 90 | 0.18 | NA |
| Boulvain, 2005 | RR | 15 | 18 | 15.0 | 85 | 0.18 | NA |
| Alfirevic, 2010 | RR | 15 | 16 | 14.4 | 85 | 0.17 | NA |
| Haas, 2008 | OR | 14 | 15 | 13.9 | 80 | 0.17 | NA |
| French, 2001 | RR | 13 | 14 | 12.9 | 75 | 0.17 | NA |

***** *Appendix S4; 11, 50, 6, 1, 4, 51, 12, 3, 39, 35*

*TE = treatment effect; N_fs_=failsafe number or numbers of hidden study(ies); benchmark = 5k+10; NA=not applicable*

**Table S5.** Results of applying the Barrowman method

| **Article identifier*** | **Measure of TE** | **Number of included studies_previous_** | **Number of included studies_updated_** | **n_actual_** | **n_expected_** | **Participant ratio** | **Predicted Status** |
| --- | --- | --- | --- | --- | --- | --- | --- |
| Crowther, 2007 | MD | 2 | 4 | 1,734 | 50 | 34.92 | out-of-date |
| Duley, 2006 | RR | 2 | 3 | 126 | 12 | 10.22 | out-of-date |
| Crepinsek, 2010 | RR | 2 | 3 | 377 | 52 | 7.25 | out-of-date |
| Doyle, 2009 | RR | 2 | 5 | 4,725 | 863 | 5.48 | out-of-date |
| Cluver, 2004 | RR | 2 | 6 | 494 | 249 | 1.98 | out-of-date |
| King, 2002 | RR | 8 | 9 | 6,241 | 3,173 | 1.97 | out-of-date |
| Rumbold, 2005 | RR | 2 | 4 | 300 | 185 | 1.62 | out-of-date |
| *Hutton, 2001* | *RR* | *2* | *3* | *116* | *143* | *0.81* | *not-out-of-date* |
| *Berghella, 2008* | *RR* | *2* | *3* | *85* | *119* | *0.71* | *not-out-of-date* |
| *Alfirevic, 2009* | *RR* | *21* | *24* | *375* | *681* | *0.55* | *not-out-of-date* |

***** *Appendix S4; 25, 32, 21, 30, 20, 52, 68, 48, 10, 4*

*TE = treatment effect*

**Table S6.** Results of applying the Ottawa method

| **Article identifier*** | **Measure of TE** | **Number of included studies_previous_** | **TE_previous_** | **p-value_previous_** | **Number of included studies_updated_** | **TE_updated_** | **p-value_updated_** | **RRR ratio** | **Predicted Status** |
| --- | --- | --- | --- | --- | --- | --- | --- | --- | --- |
| Alfirevic, 2010 | RR | 3 | 0.995 | 0.99 | 4 | 0.848 | 0.590 | 33.07 | out-of-date |
| Dare, 2006 | RR | 4 | 0.998 | 0.99 | 10 | 0.944 | 0.439 | 22.80 | out-of-date |
| Hofmeyr, 2008 | RR | 2 | 1.070 | 0.94 | 3 | 1.367 | 0.71 | 5.22 | out-of-date |
| Tooher, 2010 | RR | 2 | 1.057 | 0.94 | 3 | 1.244 | 0.73 | 4.30 | out-of-date |
| Anotayanonth, 2004 | RR | 5 | 0.953 | 0.93 | 7 | 0.803 | 0.50 | 4.14 | out-of-date |
| Porter, 2006 | OR | 11 | 1.095 | 0.66 | 12 | 1.332 | 0.28 | 3.51 | out-of-date |
| Boulvain, 2005 | RR | 15 | 0.967 | 0.82 | 18 | 0.903 | 0.44 | 2.93 | out-of-date |
| Empson, 2005 | RR | 2 | 1.021 | 0.93 | 3 | 1.054 | 0.83 | 2.54 | out-of-date |
| Brown, 2008 | RR | 5 | 0.940 | 0.42 | 7 | 0.854 | 0.07 | 2.42 | out-of-date |
| Hopkins, 1999 | OR | 10 | 0.929 | 0.81 | 11 | 0.872 | 0.55 | 1.79 | out-of-date |

***** *Appendix S4; 5, 26, 46, 74, 8, 67, 12, 33, 14, 47*

*TE = treatment effect*

**Table S7.** Results of applying the simulation-based power method

| **Article identifier*** | **Measure of TE** | **Number of included studies_previous_** | **Number of included study_info_** | **n_info-trt_** | **n_info-ctrl_** | **Power%** | **Predicted Status** |
| --- | --- | --- | --- | --- | --- | --- | --- |
| Crepinsek, 2010 | RR | 2 | 1 | 187 | 190 | 63.4 | *not-out-of-date* |
| Duley, 2006 | RR | 2 | 1 | 62 | 61 | 62.0 | *not-out-of-date* |
| Alfirevic, 2009 | RR | 21 | 3 | 178 | 197 | 60.7 | *not-out-of-date* |
| Gülmezoglu, 2006 | RR | 3 | 1 | 424 | 425 | 60.3 | *not-out-of-date* |
| Rumbold, 2005 | RR | 2 | 2 | 152 | 148 | 49.5 | *not-out-of-date* |
| Doyle, 2009 | RR | 2 | 3 | 2,338 | 2,387 | 44.7 | *not-out-of-date* |
| Cluver, 2004 | RR | 2 | 4 | 245 | 249 | 37.9 | *not-out-of-date* |
| Anotayanonth, 2004 | RR | 5 | 2 | 455 | 465 | 29.0 | *not-out-of-date* |
| King, 2002 | RR | 8 | 1 | 4,685 | 1,556 | 27.5 | *not-out-of-date* |
| Gülmezoglu, 2007 | RR | 7 | 2 | 1,263 | 1,149 | 27.0 | *not-out-of-date* |

***** *Appendix S4; 21, 32, 4, 37, 68, 30, 20, 8, 52, 38*

*TE = treatment effect;*

*Number of studyinfo = number of study(ies) published within 3 years of the most recent study;*

*ninfo-trt = number of participants in treatment arm in the study(ies) that were published within 3 years of the most recent study;*

*ninfo-ctrl = number of participants in control arm in the study(ies) that were published within 3 years of the most recent study*

**Table S8.** Strength of agreement between the methods in identifying out-of-date SRs

| **Method i** | **Method j** | **a** | **b** | **c** | **d** | **Kappa (95% CI)** |
| --- | --- | --- | --- | --- | --- | --- |
| Recursive CMA | Barrowman | 3 | 4 | 4 | 69 | **0.37 (0.03 ; 0.72)** |
| Recursive CMA | Ottawa | 4 | 3 | 30 | 43 | 0.06 (-0.09 ; 0.20) |
| Barrowman | Ottawa | 5 | 2 | 29 | 44 | 0.12 (-0.03 ; 0.26) |

*a = method i and j indicated "out-of-date";*

*b = method i indicated "out-of-date", method j indicated not "out-of-date";*

*c = method i indicated "not out-of-date", method j indicated "out-of-date";*

*d = method i and j indicated "not out-of-date"*
