# Supplementary material for: A Comparison of Statistical Methods for Identifying Out-of-Date Systematic Reviews
Source: PLoS One. 2012 Nov 20;7(11):e48894. doi: 10.1371/journal.pone.0048894 (PMC3502410; doi:10.1371/journal.pone.0048894)
Supplement: Appendix S4 — References of the cohort of CPCG reviews. (DOC) [file pone.0048894.s004.doc]

**Appendix S4**

**References of the cohort of CPCG reviews**

1. Abalos E, Duley L, Steyn DW, Henderson-Smart DJ. Antihypertensive drug therapy for mild to moderate hypertension during pregnancy. Cochrane Database of Systematic Reviews 2007, Issue 1. Art. No.: CD002252. DOI: 10.1002/14651858.CD002252.pub2.

2. Alfirevic Z, Weeks A. Oral misoprostol for induction of labour. Cochrane Database of Systematic Reviews 2006, Issue 2. Art. No.: CD001338. DOI: 10.1002/14651858.CD001338.pub2.

3. Alfirevic Z, Devane D, Gyte GML. Continuous cardiotocography (CTG) as a form of electronic fetal monitoring (EFM) for fetal assessment during labour. Cochrane Database of Systematic Reviews 2006, Issue 3. Art. No.: CD006066. DOI: 10.1002/14651858.CD006066.

4. Alfirevic Z, Kelly AJ, Dowswell T. Intravenous oxytocin alone for cervical ripening and induction of labour. Cochrane Database of Systematic Reviews 2009, Issue 4. Art. No.: CD003246. DOI: 10.1002/14651858.CD003246.pub2.

5. Alfirevic Z, Stampalija T, Gyte GML. Fetal and umbilical Doppler ultrasound in normal pregnancy. Cochrane Database of Systematic Reviews 2010, Issue 8. Art. No.: CD001450. DOI: 10.1002/14651858.CD001450.pub3.

6. Alfirevic Z, Gyte GML, Dou L. Different classes of antibiotics given to women routinely for preventing infection at caesarean section. Cochrane Database of Systematic Reviews 2010, Issue 10. Art. No.: CD008726. DOI: 10.1002/14651858.CD008726.

7. Anim-Somuah M, Smyth RMD, Jones L. Epidural versus non-epidural or no analgesia in labour. Cochrane Database of Systematic Reviews 2011, Issue 12. Art. No.: CD000331. DOI: 10.1002/14651858.CD000331.pub3.

8. Anotayanonth S, Subhedar NV, Neilson JP, Harigopal S. Betamimetics for inhibiting preterm labour. Cochrane Database of Systematic Reviews 2004, Issue 4. Art. No.: CD004352. DOI: 10.1002/14651858.CD004352.pub2.

9. Basevi V, Lavender T. Routine perineal shaving on admission in labour. Cochrane Database of Systematic Reviews 2000, Issue 4. Art. No.: CD001236. DOI: 10.1002/14651858.CD001236.

10. Berghella V, Hayes E, Visintine J, Baxter JK. Fetal fibronectin testing for reducing the risk of preterm birth. Cochrane Database of Systematic Reviews 2008, Issue 4. Art. No.: CD006843. DOI: 10.1002/14651858.CD006843.pub2.

11. Boulvain M, Stan CM, Irion O. Membrane sweeping for induction of labour. Cochrane Database of Systematic Reviews 2005, Issue 1. Art. No.: CD000451. DOI: 10.1002/14651858.CD000451.pub2.

12. Boulvain M, Kelly AJ, Irion O. Intracervical prostaglandins for induction of labour. Cochrane Database of Systematic Reviews 2008, Issue 1. Art. No.: CD006971. DOI: 10.1002/14651858.CD006971.

13. Bricker L, Neilson JP, Dowswell T. Routine ultrasound in late pregnancy (after 24 weeks' gestation). Cochrane Database of Systematic Reviews 2008, Issue 4. Art. No.: CD001451. DOI: 10.1002/14651858.CD001451.pub3.

14. Brown S, Small R, Argus B, Davis PG, Krastev A. Early postnatal discharge from hospital for healthy mothers and term infants. Cochrane Database of Systematic Reviews 2002, Issue 3. Art. No.: CD002958. DOI: 10.1002/14651858.CD002958.

15. Brown HC, Paranjothy S, Dowswell T, Thomas J. Package of care for active management in labour for reducing caesarean section rates in low-risk women. Cochrane Database of Systematic Reviews 2008, Issue 4. Art. No.: CD004907. DOI: 10.1002/14651858.CD004907.pub2.

16. Brownfoot FC, Crowther CA, Middleton P. Different corticosteroids and regimens for accelerating fetal lung maturation for women at risk of preterm birth. Cochrane Database of Systematic Reviews 2008, Issue 4. Art. No.: CD006764. DOI: 10.1002/14651858.CD006764.pub2.

17. Buchanan SL, Crowther CA, Levett KM, Middleton P, Morris J. Planned early birth versus expectant management for women with preterm prelabour rupture of membranes prior to 37 weeks' gestation for improving pregnancy outcome. Cochrane Database of Systematic Reviews 2010, Issue 3. Art. No.: CD004735. DOI: 10.1002/14651858.CD004735.pub3.

18. Carroli G, Mignini L. Episiotomy for vaginal birth. Cochrane Database of Systematic Reviews 2009, Issue 1. Art. No.: CD000081. DOI: 10.1002/14651858.CD000081.pub2.

19. Churchill D, Beevers GDG, Meher S, Rhodes C. Diuretics for preventing pre-eclampsia. Cochrane Database of Systematic Reviews 2007, Issue 1. Art. No.: CD004451. DOI: 10.1002/14651858.CD004451.pub2.

20. Cluver C, Hofmeyr GJ, Gyte GML, Sinclair M. Interventions for helping to turn term breech babies to head first presentation when using external cephalic version. Cochrane Database of Systematic Reviews 2012, Issue 1. Art. No.: CD000184. DOI: 10.1002/14651858.CD000184.pub3.

21. Crepinsek MA, Crowe L, Michener K, Smart NA. Interventions for preventing mastitis after childbirth. Cochrane Database of Systematic Reviews 2010, Issue 8. Art. No.: CD007239. DOI: 10.1002/14651858.CD007239.pub2.

22. Crowther CA, Hiller JE, Doyle LW. Magnesium sulphate for preventing preterm birth in threatened preterm labour. Cochrane Database of Systematic Reviews 2002, Issue 4. Art. No.: CD001060. DOI: 10.1002/14651858.CD001060.

23. Crowther CA, Alfirevic Z, Han S, Haslam RR. Thyrotropin-releasing hormone added to corticosteroids for women at risk of preterm birth for preventing neonatal respiratory disease. Cochrane Database of Systematic Reviews 2004, Issue 2. Art. No.: CD000019. DOI: 10.1002/14651858.CD000019.pub2.

24. Crowther CA, McKinlay CJD, Middleton P, Harding JE. Repeat doses of prenatal corticosteroids for women at risk of preterm birth for improving neonatal health outcomes. Cochrane Database of Systematic Reviews 2011, Issue 6. Art. No.: CD003935. DOI: 10.1002/14651858.CD003935.pub3.

25. Crowther CA, Han S. Hospitalisation and bed rest for multiple pregnancy. Cochrane Database of Systematic Reviews 2010, Issue 7. Art. No.: CD000110. DOI: 10.1002/14651858.CD000110.pub2.

26. Dare MR, Middleton P, Crowther CA, Flenady V, Varatharaju B. Planned early birth versus expectant management (waiting) for prelabour rupture of membranes at term (37 weeks or more). Cochrane Database of Systematic Reviews 2006, Issue 1. Art. No.: CD005302. DOI: 10.1002/14651858.CD005302.pub2.

27. Dennis CL, Creedy DK. Psychosocial and psychological interventions for preventing postpartum depression. Cochrane Database of Systematic Reviews 2004, Issue 4. Art. No.: CD001134. DOI: 10.1002/14651858.CD001134.pub2.

28. De-Regil LM, Fernández-Gaxiola AC, Dowswell T, Peña-Rosas JP. Effects and safety of periconceptional folate supplementation for preventing birth defects. Cochrane Database of Systematic Reviews 2010, Issue 10. Art. No.: CD007950. DOI: 10.1002/14651858.CD007950.pub2.

29. Dodd JM, Anderson ER, Gates S. Surgical techniques for uterine incision and uterine closure at the time of caesarean section. Cochrane Database of Systematic Reviews 2008, Issue 3. Art. No.: CD004732. DOI: 10.1002/14651858.CD004732.pub2.

30. Doyle LW, Crowther CA, Middleton P, Marret S, Rouse D. Magnesium sulphate for women at risk of preterm birth for neuroprotection of the fetus. Cochrane Database of Systematic Reviews 2009, Issue 1. Art. No.: CD004661. DOI: 10.1002/14651858.CD004661.pub3.

31. Duley L, Henderson-Smart DJ, Meher S. Drugs for treatment of very high blood pressure during pregnancy. Cochrane Database of Systematic Reviews 2006, Issue 3. Art. No.: CD001449. DOI: 10.1002/14651858.CD001449.pub2.

32. Duley L, Henderson-Smart DJ, Chou D. Magnesium sulphate versus phenytoin for eclampsia. Cochrane Database of Systematic Reviews 2010, Issue 10. Art. No.: CD000128. DOI: 10.1002/14651858.CD000128.pub2.

33. Empson MB, Lassere M, Craig JC, Scott JR. Prevention of recurrent miscarriage for women with antiphospholipid antibody or lupus anticoagulant. Cochrane Database of Systematic Reviews 2005, Issue 2. Art. No.: CD002859. DOI: 10.1002/14651858.CD002859.pub2.

34. French L. Oral prostaglandin E2 for induction of labour. Cochrane Database of Systematic Reviews 2001, Issue 2. Art. No.: CD003098. DOI: 10.1002/14651858.CD003098.

35. French L, Smaill FM. Antibiotic regimens for endometritis after delivery. Cochrane Database of Systematic Reviews 2004, Issue 4. Art. No.: CD001067. DOI: 10.1002/14651858.CD001067.pub2.

36. Grivell RM, Alfirevic Z, Gyte GML, Devane D. Antenatal cardiotocography for fetal assessment. Cochrane Database of Systematic Reviews 2010, Issue 1. Art. No.: CD007863. DOI: 10.1002/14651858.CD007863.pub2.

37. Gülmezoglu AM, Crowther CA, Middleton P. Induction of labour for improving birth outcomes for women at or beyond term. Cochrane Database of Systematic Reviews 2006, Issue 4. Art. No.: CD004945. DOI: 10.1002/14651858.CD004945.pub2.

38. Gülmezoglu AM, Forna F, Villar J, Hofmeyr GJ. Prostaglandins for preventing postpartum haemorrhage. Cochrane Database of Systematic Reviews 2007, Issue 3. Art. No.: CD000494. DOI: 10.1002/14651858.CD000494.pub3.

39. Haas DM, Ramsey PS. Progestogen for preventing miscarriage. Cochrane Database of Systematic Reviews 2008, Issue 2. Art. No.: CD003511. DOI: 10.1002/14651858.CD003511.pub2.

40. Haider BA, Bhutta ZA. Multiple-micronutrient supplementation for women during pregnancy. Cochrane Database of Systematic Reviews 2006, Issue 4. Art. No.: CD004905. DOI: 10.1002/14651858.CD004905.pub2.

41. Hatem M, Sandall J, Devane D, Soltani H, Gates S. Midwife-led versus other models of care for childbearing women. Cochrane Database of Systematic Reviews 2008, Issue 4. Art. No.: CD004667. DOI: 10.1002/14651858.CD004667.pub2.

42. Hodnett ED, Fredericks S, Weston J. Support during pregnancy for women at increased risk of low birthweight babies. Cochrane Database of Systematic Reviews 2010, Issue 6. Art. No.: CD000198. DOI: 10.1002/14651858.CD000198.pub2.

43. Hofmeyr GJ, Lawrie TA. Amnioinfusion for potential or suspected umbilical cord compression in labour. Cochrane Database of Systematic Reviews 2012, Issue 1. Art. No.: CD000013. DOI: 10.1002/14651858.CD000013.pub2.

44. Hofmeyr GJ, Kulier R. Cephalic version by postural management for breech presentation. Cochrane Database of Systematic Reviews 2000, Issue 3. Art. No.: CD000051. DOI: 10.1002/14651858.CD000051.

45. Hofmeyr GJ, Mathai M, Shah AN, Novikova N. Techniques for caesarean section. Cochrane Database of Systematic Reviews 2008, Issue 1. Art. No.: CD004662. DOI: 10.1002/14651858.CD004662.pub2.

46. Hofmeyr GJ, Gülmezoglu AM, Pileggi C. Vaginal misoprostol for cervical ripening and induction of labour. Cochrane Database of Systematic Reviews 2010, Issue 10. Art. No.: CD000941. DOI: 10.1002/14651858.CD000941.pub2.

47. Hopkins L, Smaill FM. Antibiotic prophylaxis regimens and drugs for cesarean section. Cochrane Database of Systematic Reviews 2012, Issue 1. Art. No.: CD001136. DOI: 10.1002/14651858.CD001136.pub2.

48. Hutton EK, Mozurkewich EL. Extra-amniotic prostaglandin for induction of labour. Cochrane Database of Systematic Reviews 2001, Issue 2. Art. No.: CD003092. DOI: 10.1002/14651858.CD003092.

49. Kelly AJ, Kavanagh J, Thomas J. Relaxin for cervical ripening and induction of labour. Cochrane Database of Systematic Reviews 2001, Issue 2. Art. No.: CD003103. DOI: 10.1002/14651858.CD003103.

50. Kelly AJ, Malik S, Smith L, Kavanagh J, Thomas J. Vaginal prostaglandin (PGE2 and PGF2a) for induction of labour at term. Cochrane Database of Systematic Reviews 2009, Issue 4. Art. No.: CD003101. DOI: 10.1002/14651858.CD003101.pub2.

51. Kenyon S, Boulvain M, Neilson JP. Antibiotics for preterm rupture of membranes. Cochrane Database of Systematic Reviews 2010, Issue 8. Art. No.: CD001058. DOI: 10.1002/14651858.CD001058.pub2.

52. King JF, Flenady V, Murray L. Prophylactic antibiotics for inhibiting preterm labour with intact membranes. Cochrane Database of Systematic Reviews 2002, Issue 4. Art. No.: CD000246. DOI: 10.1002/14651858.CD000246.

53. Kramer MS, Kakuma R. Optimal duration of exclusive breastfeeding. Cochrane Database of Systematic Reviews 2002, Issue 1. Art. No.: CD003517. DOI: 10.1002/14651858.CD003517.

54. Lalor JG, Fawole B, Alfirevic Z, Devane D. Biophysical profile for fetal assessment in high risk pregnancies. Cochrane Database of Systematic Reviews 2008, Issue 1. Art. No.: CD000038. DOI: 10.1002/14651858.CD000038.pub2.

55. Lavender T, Hart A, Smyth RMD. Effect of partogram use on outcomes for women in spontaneous labour at term. Cochrane Database of Systematic Reviews 2008, Issue 4. Art. No.: CD005461. DOI: 10.1002/14651858.CD005461.pub2.

56. Lawrence A, Lewis L, Hofmeyr GJ, Dowswell T, Styles C. Maternal positions and mobility during first stage labour. Cochrane Database of Systematic Reviews 2009, Issue 2. Art. No.: CD003934. DOI: 10.1002/14651858.CD003934.pub2.

57. Luckas M, Bricker L. Intravenous prostaglandin for induction of labour. Cochrane Database of Systematic Reviews 2000, Issue 4. Art. No.: CD002864. DOI: 10.1002/14651858.CD002864.

58. Lumbiganon P, Thinkhamrop J, Thinkhamrop B, Tolosa JE. Vaginal chlorhexidine during labour for preventing maternal and neonatal infections (excluding Group B Streptococcal and HIV). Cochrane Database of Systematic Reviews 2004, Issue 4. Art. No.: CD004070. DOI: 10.1002/14651858.CD004070.pub2.

59. Mahomed K, Bhutta ZA, Middleton P. Zinc supplementation for improving pregnancy and infant outcome. Cochrane Database of Systematic Reviews 2007, Issue 2. Art. No.: CD000230. DOI: 10.1002/14651858.CD000230.pub3.

60. Makrides M, Crowther CA. Magnesium supplementation in pregnancy. Cochrane Database of Systematic Reviews 2001, Issue 4. Art. No.: CD000937. DOI: 10.1002/14651858.CD000937.

61. Makrides M, Duley L, Olsen SF. Marine oil, and other prostaglandin precursor, supplementation for pregnancy uncomplicated by pre-eclampsia or intrauterine growth restriction. Cochrane Database of Systematic Reviews 2006, Issue 3. Art. No.: CD003402. DOI: 10.1002/14651858.CD003402.pub2.

62. McDonald SJ, Middleton P. Effect of timing of umbilical cord clamping of term infants on maternal and neonatal outcomes. Cochrane Database of Systematic Reviews 2008, Issue 2. Art. No.: CD004074. DOI: 10.1002/14651858.CD004074.pub2.

63. Nabhan AF, Abdelmoula YA. Amniotic fluid index versus single deepest vertical pocket as a screening test for preventing adverse pregnancy outcome. Cochrane Database of Systematic Reviews 2008, Issue 3. Art. No.: CD006593. DOI: 10.1002/14651858.CD006593.pub2.

64. Nabhan AF, Faris MA. High feedback versus low feedback of prenatal ultrasound for reducing maternal anxiety and improving maternal health behaviour in pregnancy. Cochrane Database of Systematic Reviews 2010, Issue 4. Art. No.: CD007208. DOI: 10.1002/14651858.CD007208.pub2.

65. Neilson JP. Fetal electrocardiogram (ECG) for fetal monitoring during labour. Cochrane Database of Systematic Reviews 2012, Issue 4. Art. No.: CD000116. DOI: 10.1002/14651858.CD000116.pub3.

66. Ng KW, Parsons J, Cyna AM, Middleton P. Spinal versus epidural anaesthesia for caesarean section. Cochrane Database of Systematic Reviews 2004, Issue 2. Art. No.: CD003765. DOI: 10.1002/14651858.CD003765.pub2.

67. Porter TF, LaCoursiere Y, Scott JR. Immunotherapy for recurrent miscarriage. Cochrane Database of Systematic Reviews 2006, Issue 2. Art. No.: CD000112. DOI: 10.1002/14651858.CD000112.pub2.

68. Rumbold A, Crowther CA. Vitamin C supplementation in pregnancy. Cochrane Database of Systematic Reviews 2005, Issue 1. Art. No.: CD004072. DOI: 10.1002/14651858.CD004072.pub2.

69. Singata M, Tranmer J, Gyte GML. Restricting oral fluid and food intake during labour. Cochrane Database of Systematic Reviews 2010, Issue 1. Art. No.: CD003930. DOI: 10.1002/14651858.CD003930.pub2.

70. Smyth RMD, Alldred SK, Markham C. Amniotomy for shortening spontaneous labour. Cochrane Database of Systematic Reviews 2007, Issue 4. Art. No.: CD006167. DOI: 10.1002/14651858.CD006167.pub2.

71. Soltani H, Hutchon DR, Poulose TA. Timing of prophylactic uterotonics for the third stage of labour after vaginal birth. Cochrane Database of Systematic Reviews 2010, Issue 8. Art. No.: CD006173. DOI: 10.1002/14651858.CD006173.pub2.

72. Thinkhamrop J, Hofmeyr GJ, Adetoro O, Lumbiganon P. Prophylactic antibiotic administration during second and third trimester in pregnancy for preventing infectious morbidity and mortality. Cochrane Database of Systematic Reviews 2002, Issue 4. Art. No.: CD002250. DOI: 10.1002/14651858.CD002250.

73. Thomas J, Kelly AJ, Kavanagh J. Oestrogens alone or with amniotomy for cervical ripening or induction of labour. Cochrane Database of Systematic Reviews 2001, Issue 4. Art. No.: CD003393. DOI: 10.1002/14651858.CD003393.

74. Tooher R, Gates S, Dowswell T, Davis LJ. Prophylaxis for venous thromboembolic disease in pregnancy and the early postnatal period. Cochrane Database of Systematic Reviews 2010, Issue 5. Art. No.: CD001689. DOI: 10.1002/14651858.CD001689.pub2.

75. Torvaldsen S, Roberts CL, Bell JC, Raynes-Greenow CH. Discontinuation of epidural analgesia late in labour for reducing the adverse delivery outcomes associated with epidural analgesia. Cochrane Database of Systematic Reviews 2004, Issue 4. Art. No.: CD004457. DOI: 10.1002/14651858.CD004457.pub2.

76. Wei S, Wo BL, Xu H, Luo ZC, Roy C, Fraser WD. Early amniotomy and early oxytocin for prevention of, or therapy for, delay in first stage spontaneous labour compared with routine care. Cochrane Database of Systematic Reviews 2009, Issue 2. Art. No.: CD006794. DOI: 10.1002/14651858.CD006794.pub2.

77. Whitworth M, Bricker L, Neilson JP, Dowswell T. Ultrasound for fetal assessment in early pregnancy. Cochrane Database of Systematic Reviews 2010, Issue 4. Art. No.: CD007058. DOI: 10.1002/14651858.CD007058.pub2.

78. Woudstra DM, Chandra S, Hofmeyr GJ, Dowswell T. Corticosteroids for HELLP (hemolysis, elevated liver enzymes, low platelets) syndrome in pregnancy. Cochrane Database of Systematic Reviews 2010, Issue 9. Art. No.: CD008148. DOI: 10.1002/14651858.CD008148.pub2.

79. Yamasmit W, Chaithongwongwatthana S, Tolosa JE, Limpongsanurak S, Pereira L, Lumbiganon P. Prophylactic oral betamimetics for reducing preterm birth in women with a twin pregnancy. Cochrane Database of Systematic Reviews 2005, Issue 3. Art. No.: CD004733. DOI: 10.1002/14651858.CD004733.pub2.

80. Young G, Jewell D. Interventions for leg cramps in pregnancy. Cochrane Database of Systematic Reviews 2002, Issue 1. Art. No.: CD000121. DOI: 10.1002/14651858.CD000121.
